# Sepsis-associated hyperlactatemia

**DOI:** 10.1186/s13054-014-0503-3

**Published:** 2014-09-09

**Authors:** Mercedes Garcia-Alvarez, Paul Marik, Rinaldo Bellomo

**Affiliations:** Department of Anaesthesiology, Hospital de Sant Pau, Carrer de Sant Quintí 89, Barcelona, 08026 Spain; Department of Intensive Care Medicine, Austin Hospital, Melbourne, Victoria 3084 Australia; Division of Pulmonary and Critical Care Medicine, Eastern Virginia Medical School, Norfolk, VA 23501 USA; Australian and New Zealand Intensive Care Research Centre, Melbourne, Victoria 3004 Australia

## Abstract

There is overwhelming evidence that sepsis and septic shock are associated with hyperlactatemia (sepsis-associated hyperlactatemia (SAHL)). SAHL is a strong independent predictor of mortality and its presence and progression are widely appreciated by clinicians to define a very high-risk population. Until recently, the dominant paradigm has been that SAHL is a marker of tissue hypoxia. Accordingly, SAHL has been interpreted to indicate the presence of an ‘oxygen debt’ or ‘hypoperfusion’, which leads to increased lactate generation via anaerobic glycolysis. In light of such interpretation of the meaning of SAHL, maneuvers to increase oxygen delivery have been proposed as its treatment. Moreover, lactate levels have been proposed as a method to evaluate the adequacy of resuscitation and the nature of the response to the initial treatment for sepsis. However, a large body of evidence has accumulated that strongly challenges such notions. Much evidence now supports the view that SAHL is not due only to tissue hypoxia or anaerobic glycolysis. Experimental and human studies all consistently support the view that SAHL is more logically explained by increased aerobic glycolysis secondary to activation of the stress response (adrenergic stimulation). More importantly, new evidence suggests that SAHL may actually serve to facilitate bioenergetic efficiency through an increase in lactate oxidation. In this sense, the characteristics of lactate production best fit the notion of an adaptive survival response that grows in intensity as disease severity increases. Clinicians need to be aware of these developments in our understanding of SAHL in order to approach patient management according to biological principles and to interpret lactate concentrations during sepsis resuscitation according to current best knowledge.

## Introduction

Severe sepsis and septic shock are a major health problem worldwide and among critically ill patients in particular [1,2]. Beyond the identification of the likely focus of infection and of the organisms responsible, treatment with appropriate antibiotics, and drainage of the focus itself when possible, early and aggressive hemodynamic resuscitation is recommended for the treatment of septic patients [3]. The identification of severe sepsis is based on clinical signs but also on laboratory findings. Among these, sepsis-associated hyperlactatemia (SAHL) has been recently promoted as a way of identifying patients with ‘cryptic’ shock who require focused, early goal-directed therapy [4].

In fact, SAHL is a common finding, reaching levels as high as 15.0 mmol/L in some patients [5]. Plasma lactate levels and their trend over time are reliable markers of illness severity and mortality [6,7], being recently included in a multibiomarker-based outcome risk model for adult patients with septic shock [8]. Even relative hyperlactatemia (blood lactate concentrations >0.75 mmol/L) is independently associated with increased hospital mortality [9,10].

Raised blood lactate concentrations in the setting of sepsis are frequently viewed as evidence of tissue hypoxia and/or oxygen debt secondary to hypoperfusion [11,12]. According to such paradigms, SAHL is due to anaerobic glycolysis induced by tissue hypoxia. Such tissue hypoxia in widely believed to be a major cause of organ failure and mortality. Moreover, changes in lactate concentration over time during intervention (for example, incorrectly labeled lactate ‘clearance’) have been proposed as end points in sepsis resuscitation, as means of determining the adequacy of oxygen delivery, and as indicators of resolution of global tissue hypoxia.

Despite such strongly and widely held views, the source, biochemistry, removal and metabolic functions of lactate in sepsis remain unclear. It is also uncertain whether SAHL represents a maladaptive or protective response. The sheer complexity of lactate, a ubiquitously produced and utilized metabolite that, like glucose, is central to almost every energy-related pathway in humans, is one of the main reasons for the lack of a clear understanding of its pathophysiology and clinical meaning.

In this clinical review, we examine key aspects of SAHL and explain why it cannot be used exclusively as a reliable marker of tissue hypoxia, oxygen debt or anaerobic glycolysis. Instead, we provide evidence that lactate is an important aerobically produced intermediate metabolite that is most likely released as a consequence of increased or accelerated aerobic glycolysis and the stress response. Lactate in sepsis may at times be related to tissue dysoxia but, perhaps just as frequently or even simultaneously, it may be unrelated to oxygen debt and unlikely to respond to iatrogenic increases in calculated systemic oxygen delivery. Instead, lactate may well represent an important energy source and may be helpful for survival in sepsis.

## Normal lactate metabolism

### Production

Using carbon isotopes, daily lactate production in resting humans has been estimated at approximately 20 mmol/kg/day (range of 0.9 to 1.0 mmol/kg/hour) [13]. Lactate can be released into the blood stream by many different cells [14], but the exact lactate balance (production minus utilization) at rest for each organ or tissue is unknown. Lactate clearance has been estimated at an extraordinary value of 800 to 1,800 ml/minute by studying the disposal of infused sodium L-lactate [15]. This implies that all of the blood can be cleared of lactate every 3 to 4 minutes and that, at a concentration of 1 to 2 mmol/L, 60 to 120 mmol of lactate are removed every hour.

Lactate is formed from pyruvate in the cytosol as part of glycolysis. Its concentration is in equilibrium with pyruvate as maintained by lactate dehydrogenase (LDH), an enzyme that favors lactate production and normally maintains a constant lactate to pyruvate ratio of approximately 10:1. Logically, therefore, any condition that increases pyruvate generation will increase lactate generation. Importantly, lactate generation from pyruvate releases NAD^+^, a major acceptor of electrons during glycolysis, thus potentially facilitating glycolytic energy generation. Without an efficient mechanism in the cytosol to recycle NAD^+^ from NADH, glycolysis cannot happen.

### Removal

Lactate can be metabolized by the liver and the kidney either by direct oxidation or as a source of glucose.

#### Gluconeogenesis

Its production by muscle or other tissues and its transformation into glucose by liver and kidney are known as the Cori cycle (gluconeogenesis) [16]. Lactate is quantitatively the most important gluconeogenic precursor in humans and thus a key source of glucose [17]. Hepatocytes are the major site of oxidative lactate uptake but the kidneys account for approximately 30% of lactate metabolism. Transformation of lactate into glucose by the kidneys is responsible for 50% of overall lactate conversion to glucose [18].

#### Oxidation

Lactate is not only transformed into glucose via the Cori cycle, it is also removed by oxidation (via pyruvate and the citric acid cycle) [19]. Approximately half of available lactate is disposed of via oxidation at rest, and 75 to 80% during exercise [20]. This observation suggests that lactate is a bioenergetic fuel during stress and can serve to both spare blood glucose utilization and deliver additional glucose [19]. This oxidation pathway has been carefully assessed in exercising human skeletal muscle where isotope studies confirm simultaneous lactate uptake and release by muscle [21,22]. Hyperlactatemia can cause muscle to switch from release to uptake via oxidation [23]. Myocyte compartmentalization (into a glycolytic and an oxidative compartment) has been proposed as the most logical explanation for such simultaneous lactate production and utilization in muscle [22]. The glycolytic compartment (likely close to the myofibrils and their glycogen stores) is considered associated with glycogenolysis/glycolysis and lactate release. The oxidative compartment (likely close to the mitochondria) is considered responsible for lactate uptake/oxidation. This 'intracellular lactate shuttle' hypothesis implies that lactate production via glycolysis in the cytosol is balanced by oxidation in the mitochondria of the same cell [24].

**Figure 1:**
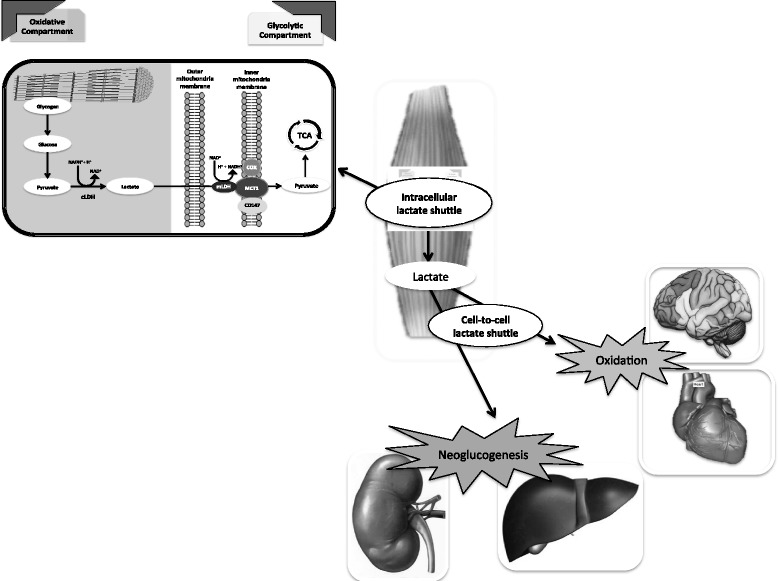
**Schematic view of the intracellular lactate shuttle with the mitochondrial lactate oxidation complex and the cell-to-cell lactate shuttle (CCLS).** Myocytes have a glycolytic and an oxidative compartment. The glycolytic compartment in the cytosol is close to the myofibrils and their glycogen stores. It is associated with glycogenolysis/glycolysis and lactate release into the circulation. The oxidative compartment in close proximity to the mitochondria is considered responsible for lactate oxidation. Lactate produced in the cytosol is oxidized to pyruvate via the lactate oxidation complex in the mitochondria of the same cell. Pyruvate is then transported across the inner mitochondrial membrane via a monocarboxylate transport protein (MCT1). MCT1 is found in the mitochondrial inner membrane as part of the lactate oxidation complex together with its chaperone protein CD147, cytochrome oxidase (COX) and mitochondrial lactate dehydrogenase (mLDH). mLDH is found in the outer side of the inner membrane. Once pyruvate enters the mitochondrial matrix, it is metabolized by the tricarboxylic acid cycle (TCA). The CCLS hypothesis supports the idea that lactate produced in muscle can also serve as a substrate in highly oxidative cells (heart, brain) or contribute to gluconeogenesis (liver, kidney). cLDH, cytosolic lactate dehydrogenase.

Contrary to past beliefs that lactate is confined to the cytosol, recent evidence has also clearly demonstrated that lactate produced in the cytosol can be transported across mitochondria by monocarboxylate transport proteins (MCTs) and oxidized to pyruvate by a mitochondrial lactate oxidation complex (mLOC). This mLOC is composed of a mitochondrial LDH, a transmembrane glycoptrotein called CD147, acting as the chaperone protein for MCT type 1, and also a cytochrome oxidase. This complex is localized at the level of the mitochondrial inner membrane, as demonstrated by confocal laser scanning microscopy, western blotting of cell subfractions, and immunoprecipitation techniques [24]. Low concentrations of lactate in the mitochondrial matrix facilitate lactate mitochondrial influx and oxidation to pyruvate. Pyruvate is then transported from the intermembrane space where mLOC resides, into the mitochondrial matrix and oxidized via the tricarboxylic acid cycle [25,26]. Importantly, MCT1 and mLOC-related genes are differentially upregulated by lactate through a positive feedback loop [27]. Increasing expression of lactate transporters on mitochondrial membranes allows a more effective 'intracellular lactate shuttle'. Some lactate can also be exported to adjacent cells, tissues and organs to serve as oxidative or gluconeogenic substrates as part of an adjacent ‘cell-to-cell lactate shuttle’ [26] (Figure 1).

## Lactate use during stress

The heart takes up and oxidizes lactate at rest [28]. Myocardial lactate uptake increases during exercise, β-adrenergic stimulation, elevated afterload, fast pacing and during shock [29–31]. During hyperlactatemia, lactate can account for up to 60% of cardiac oxidative substrate and exceed glucose as a source of pyruvate [30]. During shock, the heart oxidizes lactate for the majority of its energy needs [29]. Lactate infusion increases cardiac output in anesthetized pigs and cardiac performance in patients with acute heart failure [32] and both cardiogenic and septic shock [33]. Indeed systemic lactate deprivation is associated with cardiovascular collapse and early death of the animals [33,34].

The human brain changes to a lactate consumer during increased metabolic demand [35]. Lactate accounts for about 7% of cerebral energy requirement under basal conditions and up to 25% during exercise [14]. Blood lactate is oxidized by neurons in the conscious healthy human brain or converted to glycogen in astrocytes. The contribution of lactate as a brain energy source increases during hyperlactatemia [35,36]. Lactate is used as a primary energy source during experimental insulin-induced hypoglycemia and is readily oxidized by the brain in an activity-dependent manner [37]. Collective evidence from brain-functional imaging literature supports the existence of an ‘astrocyte-neuronal lactate shuttle’ where lactate derived from astrocyte glycolysis is transported to adjacent neurons, converted to pyruvate and oxidized via the tricarboxylic acid cycle [38].

## Why has the tissue hypoxia paradigm emerged and dominated until now?

The clinical syndrome of lactic acidosis was popularized by Huckabee and Weil over five decades ago [39,40]. These authors proposed that elevated blood lactate levels during experimental and clinical shock states served as a measure of the degree of oxygen deficit and the severity of injury. It became widely believed that, in critically ill patients, when oxygen delivery failed to meet oxygen demand an oxygen debt with global tissue hypoxia and lactic acidosis would ensue [12,41,42]. Furthermore, classic teaching describes type A lactic acidosis, which occurs due to inadequate oxygen delivery with the presence of anaerobic glycolysis, and type B lactic acidosis, which occurs in the absence of anaerobic glycolysis and is secondary to altered clearance, malignancy, or drugs [42]. It is widely assumed that type A lactic acidosis is the cause of an elevated lactate concentration in the critically ill patient with an overt or occult hemodynamic disturbance [12,41,42]. An increased blood lactate concentration is therefore regarded as evidence of anaerobic metabolism and tissue hypoxia. It follows from this reasoning that patients with an elevated blood lactate level should be treated by increasing oxygen delivery.

## Source of lactate in sepsis

The physiologic source of lactate generation during sepsis is currently a matter of debate and research. Recent data suggest that other potential non-hypoxic causes could contribute to SAHL.

Human studies have often failed to show a relationship between hyperlactatemia and any indicators of tissue hypoxia or other indices of impaired cellular oxygenation (oxygen delivery/oxygen extraction; Table 1).

**Table 1:** **Lack of evidence for the 'traditional' mechanisms explaining sepsis-associated hyperlactatemia**

**Tissue hypoxia**		
Boekstegers *et al.* [43]	Muscle PO_2_ in septic patients	No evidence of muscle hypoxia
Sair *et al.* [44]		
Levy *et al.* [45]		
VanderMeer *et al.* [46]	Intestinal and bladder mucosal PO_2_ in septic animals	No evidence of mucosal hypoxia
Rosser *et al.* [47]		
Hotchkiss and Karl [48]	Cellular oxygenation by using hypoxic marker ([^18^ F] fluoromisonidazole) in septic animals	No cellular hypoxia in muscle, heart, lung and brain
Regueira *et al.* [49]	Measurements of HIF-1α in septic patients/animals	No relation between HIF-1α and lactate levels
Textoris *et al.* [50]		
Opdam and Bellomo [51]	Lactate production by the lung in septic shock patients	Substantial lactate release by the lung
Mitochondrial dysfunction		
Hotchkiss and Karl [48]	Measurements of ATP and PCr in muscle samples of septic animals/patients	No decrease in any of the indicators of mitochondrial function
Alamdari *et al.* [53]		
Brealey *et al.* [54]		
Pyruvate dehydrogenase		
Alamdari *et al.* [53]	Mitochondrial PDH activity in septic animals/patients	No association between PDH deficit/dysfunction and lactate increase
Jahoor *et al.* [55]		
Stacpoole *et al.* [56]		
		Dichloroacetate lowers lactate levels by stimulating the PDH complex
DO_2_ – VO_2_ mismatch		
Ronco *et al.* [57,58]	Critical DO_2_ in septic patients as they approached death	No association between hyperlactatemia and decreased DO_2_ or impaired O_2_ER
Mira *et al.* [59]	Relationship between DO_2_/SvO_2_ and SAHL	No relationship between DO_2_/SvO_2_ was found
Astiz *et al.* [60]		
Marik and Sibbald [65]		Increases in DO_2_ did not decrease lactate concentration in SAHL

DO_2_, oxygen delivery; HIF, hypoxia-inducible factor; O_2_ER, oxygen extraction ratio; PCr, phosphocreatine; PDH, pyruvate dehydrogenase; PO_2,_ partial pressure of oxygen; SAHL, sepsis-associated hyperlactatemia; SvO_2_, mixed venous oxygen saturation.

### Tissue hypoxia

Boekstegers and colleagues [43] measured bicep muscle partial pressure of oxygen (PO_2_) by intermittent and continuous methods in 70 patients distributed in three different groups (sepsis, limited infection and cardiogenic shock). These investigators found normal tissue PO_2_ values in all groups and elevated values in the septic group (reaching levels as high as 50 mmHg in the severe septic state). No correlation between serum lactate levels and muscle PO_2_ was found. Even in patients in the final state of hypodynamic septic shock leading to death, mean muscle PO_2_ did not decrease to <30 mmHg. Considering that normal muscle PO_2_ values vary from 15 to 30 mmHg, it is difficult to imagine that muscle hypoxia could explain SAHL in these patients.

Sair and colleagues [44] compared the values of forearm muscle PO_2_ and subcutaneous PO_2_ between severe septic patients and healthy volunteers. They found increased muscle oxygenation in the septic group despite a mean plasma lactate concentration of 2.8 ± 0.4 mmol/L in the septic group. These investigators also measured forearm blood flow by plethysmography. No statistical differences were found between both groups. More recently, Levy and colleagues [45] used a microdialysis technique to measure tissue PO_2_ in the quadriceps femoris muscle in patients with septic shock. They found values >36 mmHg in all patients despite raised blood lactate concentrations (4.0 ± 2.1 mmol/L) at the time of the study.

Lack of tissue hypoxia during SAHL was demonstrated in other tissues apart from the muscle. Intestinal and bladder mucosal PO_2_ values have been also reported to actually increase during sepsis in animal experiments [46,47]. Hotchkiss and Karl [48] assessed the adequacy of cellular oxygenation in sepsis by using [^18^ F] fluoromisonidazole (an hypoxic marker). These investigators found no evidence of cellular hypoxia in muscle (gastrocnemius and diaphragm), heart, lung and brain despite an increase in lactate concentration in septic animal models compared with non-septic controls. Regueira and colleagues [49] showed in septic animals that hypoxia-inducible factor (HIF)-1α was not expressed in the skeletal and cardiac muscle, pancreas, lung, or kidney despite a doubling in lactate levels. They also found that the respiration of skeletal muscle and liver-isolated mitochondria was normal.

In humans, the HIF-1α mRNA concentration was measured in patients with shock (septic, hemorrhagic or cardiogenic) and compared with that of a normal group. An increased expression was found in patients with shock compared to controls. However, investigators did not find any relationship between HIF-1α expression and lactate levels, outcomes or tissue oxygenation [50].

Opdam and Bellomo [51] found substantial lactate release from the lungs of patients with septic shock. It is biologically implausible that the lungs, bathed in oxygen and receiving the full cardiac output, would experience hypoperfusion, tissue hypoxia or anaerobic metabolism.

### Mitochondrial dysfunction

A mitochondrial defect in oxygen utilization has been suggested as an explanation for SAHL in the presence of high tissue PO_2_. The concentrations of high-energy phosphates such as ATP or phosphocreatine (PCr) and the intracellular cytosolic pH are sensitive indicators of mitochondrial function and can be used to test for such postulated bioenergetic failure. Different animal studies assessed muscle metabolism in sepsis by using phosphorus 31 nuclear magnetic resonance spectroscopy. These studies showed no evidence of alterations in high-energy phosphate metabolism (normal values of ATP were found with reduced values of PCr, which acts as a reservoir for ATP). No decrease in intracellular cytosolic pH was found in any of the studies [48,52].

Alamdari and colleagues [53] found no change in ATP and PCr concentrations in animal muscle samples once sepsis was induced compared with a control group. Muscle lactate concentration was greater in the septic group compared with controls. Brealey and colleagues [54] analyzed ATP concentrations in skeletal muscle biopsies of patients with sepsis to compare these with a control group. Although lower ATP concentrations were found in non-surviving versus surviving septic patients, values remained higher in surviving septic patients compared with the control group.

### Pyruvate dehydrogenase

Mitochondrial pyruvate dehydrogenase (PDH) is an enzyme complex that regulates the conversion of pyruvate into acetyl-coenzyme A (CoA) in the mitochondria. PDH function has been reported to be impaired in sepsis and as another possible explanation for SAHL (sepsis-induced PDH dysfunction). However, investigators using tracer techniques in patients with sepsis have not found a deficit but rather an increase in PDH activity and increased glycolytic flux to oxidation [55]. In animals there was no reduction of muscle PDH activity in the early phase of muscle lactate increases during sepsis. However, after 24 hours, inhibition of the PDH complex exists possibly due to an inflammatory up-regulation of pyruvate dehydrogenase kinase (enzyme part of the PDH complex that decreases pyruvate flux through the PDH complex), thus limiting the conversion of pyruvate into acetyl-CoA [53]. If these changes apply to humans and PDH function is decreased in sepsis, then pyruvate will accumulate. This, in turn, will increase lactate production, without any need to invoke tissue hypoxia.

In addition, dichloroacetate (DCA), a drug that stimulates PDH complex activity, lowers lactate levels in septic patients and increases the rate of pyruvate oxidation and yet has no effect on tissue PO_2_. Numerous animal models and human studies have shown that DCA decreases intracellular lactate concentrations and has a dose-dependent hypolactatemic effect in septic states [56]. As DCA has no effect on tissue oxygenation, such observations challenge the hypoxia paradigm of SAHL.

### DO_2_-VO_2_ mismatch

A mismatch between tissue-level oxygen delivery (DO_2_) and oxygen consumption (VO_2_) has been proposed as an explanation for SAHL. Thus, indicators of tissue perfusion/oxygenation (cardiac index, DO_2,_ VO_2,_ oxygen extraction ratio (O_2_ER), central/mixed venous oxygen saturation (cSvO_2_/SvO_2_)) have been assessed in relation to lactate blood levels.

Ronco and colleagues [57] tried to identify the critical DO_2_ in ICU patients (including septic patients) as they approached death. A raised arterial lactate concentration was not associated with decreased DO_2_ or impaired tissue O_2_ER. In fact, baseline DO_2_ in these patients was about three times the critical DO_2_ in the presence of a lactate concentration of 4.2 ± 3.2 mmol/L. There was also no difference in critical DO_2_ between patients who had normal or increased arterial lactate values. In a further study, these investigators found that, in septic patients, there was no clinical difference in the relationship between DO_2_ and VO_2_ (measured by expired gas analysis) between patients who had normal or increased plasma lactate concentrations [58]. Mira and colleagues [59] confirmed these findings in patients with severe sepsis and SAHL.

**Figure 2:**
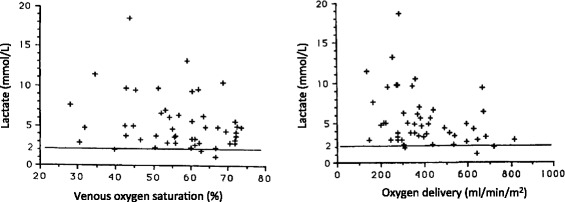
**Relationship between arterial blood lactate levels and oxygen delivery (DO**_**2**_ **)/mixed venous oxygen saturation (SvO**_**2**_ **)**_**.**_ No critical values of DO_2_ or SvO_2_ were seen to be associated with hyperlactatemia in septic patients (mean values of lactate 5.3 mmol/L). Increases in arterial lactate concentrations were present over a wide range of DO_2_ and SvO_2_ values.

Astiz and colleagues [60] were also unable to identify a critical DO_2_ or SvO_2_ associated with increased lactate concentrations in septic patients (mean value of 5.3 ± 0.5 mmol/L), even after a separate analysis of those patients who ultimately died. Increases in arterial lactate concentrations were present over a wide range of DO_2_ and SvO_2_ values (Figure 2).

The premise of increasing oxygen delivery in patients with sepsis is based on the assumption that sepsis is a hypermetabolic condition with patients having an imbalance between oxygen supply and demand as indicated by an increased lactate concentration [12,41,42]. In patients with sepsis, however, oxygen consumption and energy expenditure are broadly comparable to those of normal people, with energy expenditure actually decreasing with increasing sepsis severity [61–63]. Therefore, there is no requirement that oxygen delivery increase with sepsis. Increasing oxygen delivery in patients without an oxygen debt will not increase oxygen consumption and is likely to be harmful. Hayes and colleagues [64] performed a randomized controlled trial in which patients were randomized to ‘supranormal oxygen delivery’ or standard therapy. Despite a significant increase in oxygen delivery in the supranormal group, oxygen consumption remained unchanged while the mortality was significantly higher than in the control group. Similarly, Marik and Sibbald [65] demonstrated that blood transfusion in patients with SAHL did not increase measured oxygen consumption or result in a decrease in lactate concentration.

Finally, and more strikingly, a recent randomized clinical trial studied the hemodynamic effect of esmolol infusion in patients with septic shock. Esmolol induced a significant decrease in SAHL (*P* = 0.006) compared with placebo, even though it simultaneously reduced oxygen delivery (*P* < 0.001) [66]. If inadequate perfusion/oxygenation was the cause of hyperlactatemia, maneuvers to increase systemic or regional oxygen transport to supranormal values should correct hyperlactatemia; in this study the opposite was true. In addition, no studies have ever demonstrated such an effect. Finally, if, as appears obvious from the above observations, SAHL is not a consequence of lack of oxygen, another explanation needs to be considered.

## Alternative explanations for sepsis-associated hyperlactatemia

### Adrenergic-driven aerobic glycolysis

Accelerated aerobic glycolysis induced by sepsis-associated inflammation has been proposed as a more likely explanation for SAHL. In other words, SAHL represents a change in metabolic state, not a response to cell oxygenation issues. This theory holds that an altered metabolic state occurs when the rate of carbohydrate metabolism exceeds the oxidative capacity of the mitochondria. Pyruvate is produced by an increased utilization of glucose. Pyruvate is thus produced faster than it can be transformed into acetyl CoA by PDH. This increases cellular pyruvate concentration, which in turn increases lactate production by a mass effect. This theory is simple and logical. However, it is important to assess what observations support it.

First, preliminary data obtained from whole blood mRNA analysis in septic patients suggest significantly increased gene expression of enzymes and membrane transporters associated with glycolytic and lactate metabolism, namely glucose transporter (GLUT-1), hexokinase-3, pyruvate kinase (PKM-2), subunit A of LDH and MCT4 (unpublished data).

Second, isotope dilution methods show that, in severe sepsis, the turnover of both glucose and lactate is increased. Insulin resistance as seen in sepsis also favors glycolysis and glucose-lactate cycling. Crucially, in severe sepsis, hyperlactatemia appears related to increased production whereas lactate removal is similar to that of healthy subjects as shown by Revelly and colleagues [33], who studied lactate kinetics in patients with severe sepsis.

Pyruvate concentration is also increased by increased protein catabolism (sepsis-induced muscle proteolysis) as shown by an increase in the mRNA of proteolytic genes in skeletal muscle. This causes a release of amino acids like alanine, which is subsequently transformed into pyruvate by alanine aminotransferase and thereafter into lactate [67].

Endogenous/exogenous catecholamines are highly correlated with hyperlactatemia in sepsis. Through β_2_-receptor stimulation they increase the activity of the Na^+^/K^+^-ATPase pump [68]. Human and animal studies have demonstrated that epinephrine increases lactate formation by an increase in the Na^+^/K^+^-ATPase activity [45,69]. Levy and colleagues [70] confirmed this using a selective β_2_-blockade and muscle microdialysis in a model of endotoxic shock. If beta-adrenergic activity is responsible to a clinically relevant degree for SAHL then, logically, in humans as in animal models one might expect that beta-blockade would simultaneously decrease oxygen delivery and yet also decrease lactate concentrations. No good quality human data are available to confirm or refute this implication of the metabolic theory of SAHL in humans [66].

**Figure 3:**
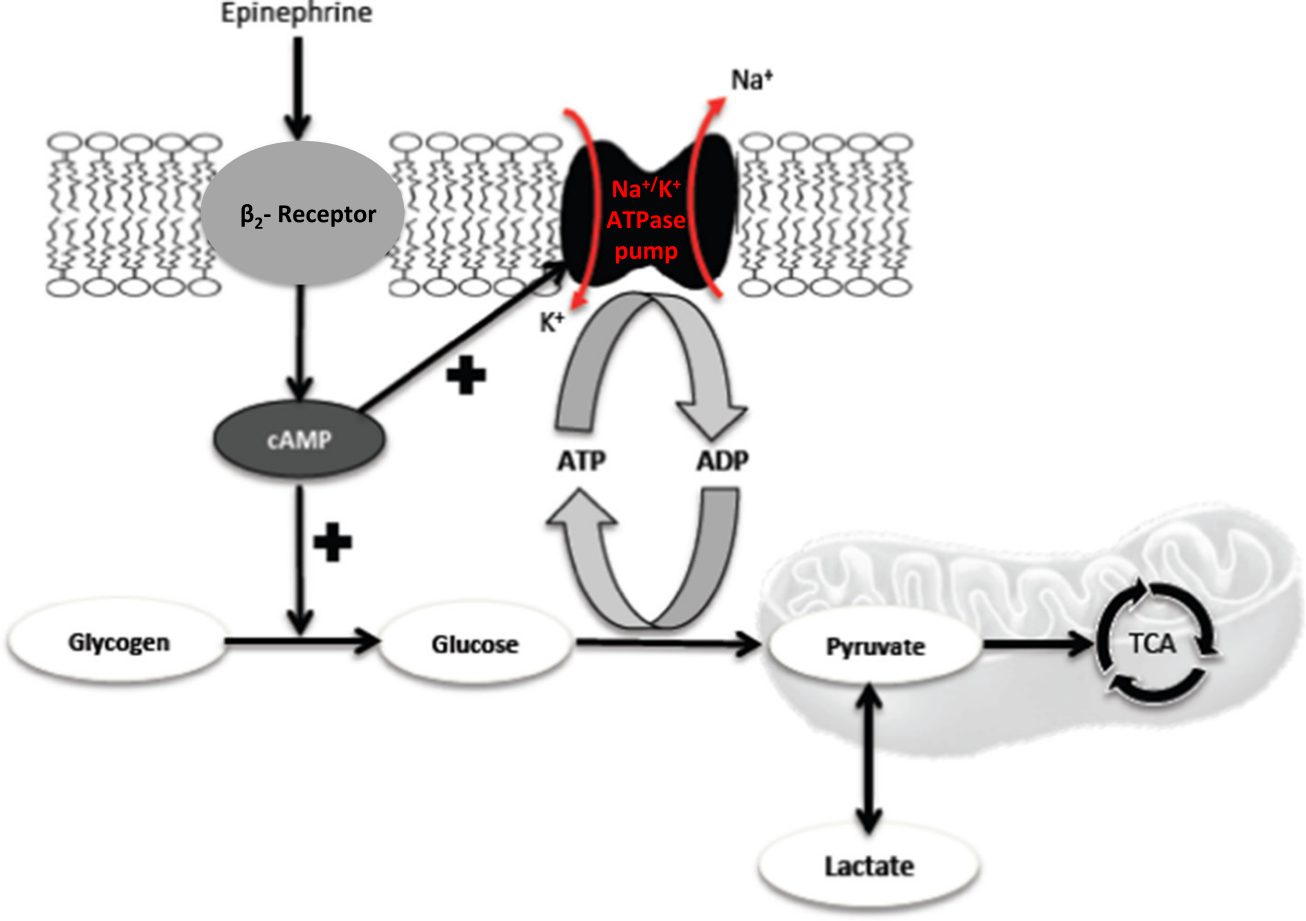
**Epinephrine-increased glycogenolysis and glycolysis is coupled to a Na** ^**+**^ **/K** ^**+**^ **-ATPase pump.** Epinephrine increases cyclic AMP (cAMP) production, inducing stimulation of glycogenolysis/glycolysis and activation of the Na^+^/K^+^-ATPase pump. This activation consumes ATP, leading to the generation of ADP. ADP reactivates glycolysis and hence generates more pyruvate and, consequently, more lactate. TCA, tricarboxylic acid cycle.

There are also logical biochemical explanations as to how adrenergic stimulation might increase lactate in sepsis. Epinephrine increases cyclic AMP, thereby inducing stimulation of glycogenolysis and glycolysis with concomitant production of ATP and activation of the Na^+^/K^+^-ATPase pump. This activation consumes ATP, leading to the generation of ADP. ADP, via phosphofructokinase stimulation, reactivates glycolysis and hence generates more pyruvate and, consequently, more lactate (Figure 3).

Moreover, the role of Na^+^/K^+^-ATPase pump stimulation was further confirmed by Levy and colleagues [45] when muscle lactate production was totally inhibited by ouabain. Of clinical importance, in patients with shock, the ability to increase glycolysis and lactate production upon epinephrine stimulation is associated with better prognosis [71], suggesting that this is an adaptive response.

## Where does lactate come from in sepsis?

Only limited information exists about the source of lactate in sepsis. This is because obtaining such information would require the invasive cannulation of major veins (renal, hepatic, portal, femoral, jugular, pulmonary) in order to measure lactate fluxes across vital organs and determine whether a particular organ adds or removes lactate during sepsis.

Investigators, however, using experimental models found the lung to be the major source of lactate [72]. Indeed, the lung changed from uptake to lactate production after induction of endotoxemia. Muscle and liver lactate fluxes were neutral and lactate uptake occurred in the gut and kidneys before and after endotoxemia. These investigators also reported that lactate is taken up by both gut and kidney during sepsis to a degree that is closely correlated with organ VO_2_, a finding consistent with the metabolic theory of lactate as an obligatory additional oxidation substrate during stress.

More directly relevant, in patients with septic shock, the lungs are a major source of lactate in a manner similar to animal models with an estimated total lactate release rate of 55.4 mmol/hour (interquartile range 24.3 to 217.6) [51]. Using continuous infusion of isotopic lactate and pyruvate, one can determine that the lungs simultaneously extract and release lactate, and that epinephrine stimulates lung conversion of pyruvate to lactate and lactate release into the systemic circulation [73].

Levy and colleagues [45] found that lactate and pyruvate concentrations measured by microdialysis are higher in muscle than in arteries (muscles are 40% of total cell mass) during septic shock. Muscles could, therefore, also have an important role in lactate production.

De Backer and colleagues [74] demonstrated that the splanchnic region is an uncommon source of net lactate generation in septic patients, even when arterial lactate concentrations are very high. Indeed, in sepsis, the splanchnic area consumes lactate rather than producing it.

In general, although not specifically studied in sepsis, the brain seems to be a major consumer rather than a lactate producer. As shown in critically ill patients before and after liver transplantation with or without hyperlactatemia, there is a net lactate uptake by the brain [75]. During sepsis the heart changes its metabolic substrate. It shifts from using free fatty acids to increased lactate utilization. Thus, the heart removes lactate.

If the splanchnic bed consumes lactate, if muscle is not hypoxic, if brain and heart consume lactate during stress, if the kidney uses lactate for the Cori cycle and if lung releases lactate during sepsis, it is difficult to conceive of any organ that is both hypoxic, underperfused, and in a state of anaerobic glycolysis responsible for lactate release.

Labeled exogenous lactate studies in septic patients show that oxidation by cells is the major fate (50 to 60%) of infused lactate. This further supports the notion that hyperlactatemia represents an adaptive protective mechanism by favoring lactate oxidation as an energy source. The amount of lactate not oxidized or converted into plasma glucose, however, remains substantial (approximately 30%) and becomes a substrate for glycogen synthesis by the liver and the kidney. Thus, under stress, lactate acts as an alternative fuel to glucose and a source of glucose itself.

## The concept of lactate clearance

In 2004 Nguyen and colleagues reported that 'lactate clearance', defined as the percentage decrease in lactate from emergency department presentation to 6 hours later, was an independent predictor of mortality [41]. They concluded that 'lactate clearance in the early hospital course may indicate a resolution of global tissue hypoxia and that this is associated with decreased mortality rates*.*' This study popularized the concept of 'lactate clearance'.

Jones and colleagues [76] extended the concept of targeting resuscitation in sepsis to achieve a lactate ‘clearance’ of at least 10% as a marker of restoration of oxygen delivery to the tissues with resuscitation treatment. However, as mentioned above, we lack evidence to justify any assumption that this fall is due to a correction of an oxygen debt. The most recent Surviving Sepsis Campaign guidelines recommend 'targeting resuscitation to normalize lactate in patients with elevated lactate levels as a marker of tissue hypoperfusion' (grade 2C) [12].

In this sense, the concept of 'lactate clearance' is misleading and should not be logically used in patients with sepsis as either the final decision point in the resuscitation strategy to determine adequacy of oxygen delivery or a target for interventions (additional management to normalize lactate clearance). Further, the term ‘clearance’ in relation to lactate is scientifically and pharmacokinetically incorrect. Clearance represents the removal of a substance from a unit of volume over a unit of time, typically expressed in milliliters per minute. Logically, it is impossible to know if the rate and/or amount of decline in SAHL is due to increased removal, decreased production, dilution because of fluid administration during resuscitation or all the above in variable combinations. Moreover, increased lactate production can remain concealed by increased utilization in septic patients, suggesting that a normal blood level of lactate does not prove that its metabolism is normal [33].

### The argument in favor of tissue hypoxia as the cause of sepsis-associated hyperlactatemia

In this review, we have made a strong case in favor of the ‘adrenergic/metabolic/energy optimization’ theory of SAHL and have provided a critique of the ‘dysoxia/tissue hypoxia/tissue hypoperfusion/anaerobic glycolysis’ theory of SAHL. We have taken this approach because we felt that, for historical reasons, the traditional view was dominant and required challenge, if only to trigger further research and reflection. However, much evidence also supports the view that tissue hypoxia may indeed occur in many patients with sepsis and be a major trigger for SAHL. First, there are many experimental and human studies linking measures of oxygen delivery and oxygen consumption to hyperlactatemia [77–80]. Such studies make a strong circumstantial case for lactate as a marker of dysoxia. Second, studies measuring the lactate/pyruvate ratio in sepsis have shown this to be increased [80–82]. Such an increased ratio provides additional evidence that tissue hypoxia may exist and may be relatively frequent in the setting of SAHL. Third, more recent work has highlighted the importance of the microcirculation in sepsis [83,84]. Such studies show profound derangements of the microcirculation in sepsis with areas of no flow or slow flow or overly fast flow [84,85]. Such abnormalities of micro-regional flow can easily and logically be seen as likely to impair oxygen delivery at a cellular level. Indeed, oxygen desaturation at a venular level is seen under these circumstances [84]. Thus, the tissue hypoxia theory presents a strong case, similar in strength to the metabolic theory presented above.

## Conclusion

Hyperlactatemia is common in patients with sepsis, a marker of illness severity and a strong predictor of mortality. However, in this review, we critique the theory that SAHL indicates an oxygen debt or hypoperfusion or tissue hypoxia or ‘anaerobic glycolysis’. We provide evidence that metabolic changes can account for SAHL and that such evidence is recurrent, logical and consistent and not yet contradicted by any empirical observation. SAHL may thus reflect severity of illness and the degree of activation of the stress response (and release of epinephrine). If the metabolic theory of SAHL is correct, then in a metaphorical sense SAHL may be the cellular equivalent of fever and may represent the impact of major changes in numerous metabolic processes. Under stress, lactate is a source of energy in the same cell where it is produced and also in other cells where it can be used as an important fuel for oxidation and glucose generation. Fluid resuscitation- or hemodynamic-based protocols may not directly affect lactate if the mechanisms of its production are not directly targeted by such activities. Similarly, lactate may not necessarily indicate the need to deliberately increase calculated systemic oxygen delivery because it may not represent an oxygen deficiency. In contrast, if the tissue hypoxia theory of SAHL is correct, then the therapeutic implications are very different. It is possible, maybe likely, that both (tissue hypoxia and metabolic adaptation) explain SAHL in different patients at different times or occur simultaneously to a degree that changes from patient to patient and according to illness severity, genetics and interventions, in a way that we do not yet understand. The extraordinary complexity of lactate makes it impossible, at this stage, to achieve such deeper understanding. Until then, clinicians should be aware of such complexity and make therapeutic choices on the basis of such knowledge.

## Abbreviations

CoA: Coenzyme A
DCA: Dichloroacetate
DO_2_: Oxygen delivery
HIF: Hypoxia-inducible factor
LDH: Lactate dehydrogenase
MCT: Monocarboxylate transport protein
mLOC: mitochondrial lactate oxidation complex
O_2_ER: Oxygen extraction ratio
PCr: Phosphocreatine
PDH: Pyruvate dehydrogenase
PO_2_: Partial pressure of oxygen
SAHL: Sepsis-associated hyperlactatemia
SvO_2_: Mixed venous oxygen saturation
VO_2_: Oxygen consumption
